# Frailty assessment to individualize treatment in older patients with lymphoma

**DOI:** 10.1007/s41999-023-00870-2

**Published:** 2023-10-12

**Authors:** Ana I. Hormigo-Sanchez, Alberto Lopez-Garcia, Ignacio Mahillo-Fernandez, Elham Askari, Daniel Morillo, María A. Perez-Saez, Miriam Riesco, Celia Urrutia, Francisco J. Martinez-Peromingo, Raúl Cordoba, Juan I. Gonzalez-Montalvo

**Affiliations:** 1 Department of Geriatric Medicine, Fundación Jiménez Díaz University Hospital, Avenida Reyes Católicos, 2. CP 28040, Madrid, Spain; 2 Department of Hematology, Fundación Jiménez, Diaz University Hospital, Madrid, Spain; 3 Department of Geriatric Medicine, La Paz University Hospital, Madrid, Spain; 4 Department of Biostatistics, Fundación Jiménez, Diaz University Hospital, Madrid, Spain; 5 General Director of Sociosanitary Coordination, Community of Madrid, Madrid, Spain; 6 Oncohealth Institute, Fundación Jiménez Diaz University Hospital, Madrid, Spain; 7 Health Research Institute IIS-FJD, Madrid, Spain; 8 Faculty of Medicine, Department of Medicine, Autonoma University of Madrid, Madrid, Spain; 9 Biomedical Research Institute of La Paz University Hospital. IdiPAZ, Madrid, Spain

**Keywords:** Lymphoma, Comprehensive geriatric assessment, Frailty, Geriatric care, Oncogeriatric, Hematologic neoplasm

## Abstract

**Purpose:**

A study analyzing the application of a protocol of comprehensive geriatric assessment (CGA) in older patients with lymphoma was carried out to allow frailty-based patient classification and individualized treatment.

**Methods:**

Lymphoma patients older than 70 years referred to the Geriatric Clinic at a tertiary hospital between May 2016 and March 2021 were included. The assessment protocol included comorbidity, polypharmacy, nutritional, functional, and mental status, geriatric syndromes, and life expectancy. CGA enabled patient classification into four groups (Type I to Type IV) based on frailty assessment instrument scoring and clinical, functional, and mental status. Variables were compared using parametric and non-parametric statistical tests and Kaplan–Meier survival curves.

**Results:**

Ninety-three patients (55.9% women) were included. Median age was 81.1 years (± 5.7). 23 patients (24.7%) were classified as robust (type I), 30 (32.3%) as pre-frail (type II) with potentially reversable deficits, 38 (40.9%) as frail (type III), and 2 (2.2%) as requiring palliative care (type IV). Patients received oncospecific treatment with modifications carried out in 64.5% of cases based on CGA results. Differences in overall survival (*p* = 0.002), response to treatment (*p* < 0.001) and likelihood of increased frailty (*p* = 0.024) were observed, with type III–IV patients showing significantly worse outcomes.

**Conclusion:**

Performance of standardized, systematic CGA by geriatricians permits older lymphoma patients to be classified according to frailty, with significant differences in terms of clinical outcomes across groups. We propose incorporating CGA performed by geriatricians as part of the multidisciplinary care team to optimize therapeutic strategy for these patients.

## Key summary points

**Aim:**

To study the application of a systematic protocol for specialized CGA in patients with lymphoma over 70 years of age.

**Findings:**

Patients were classified by level of frailty, with different groups showing statistically significant differences in overall survival, response to treatment, and likelihood of increased frailty at the end of treatment.

**Message:**

This study suggests that standardized, systematic CGA performed by geriatricians permits patient classification by level of frailty, helps in decision-making, and predicts clinical outcomes.

## Introduction

In industrialized countries, around 45% of new hematological malignancy (HM) diagnoses are made in patients aged 75 years or older [1]. HMs comprise a spectrum of different diseases, of which lymphomas are the most common; non-Hodgkin’s lymphoma (NHL) encompasses more than 20 subtypes of lymphoid diseases, classified by morphological, cytogenetic and immunophenotypic characteristics. NHL mainly affects the older population, with a median age at diagnosis of 67 years [2]. Its incidence and mortality rank 12th worldwide according to GLOBOCAN 2020 [3], with the highest incidence observed in Australia and New Zealand, Northern America, Northern Europe, and Western Europe (> 10/100,000 inhabitants for both sexes combined). In Spain, the incidence of NHL is 7.5–9.1/100,000 inhabitants for both sexes combined.

Older patients with HMs are characterized by decreased physiological reserve, leading to reduced treatment tolerance, and complicating the diagnostic and therapeutic decision-making process. Although chronological age itself is not an accurate marker of an individual patient’s biological situation and should not be used as a discriminatory variable when deciding on a therapeutic option [4], older patients with HM often present age-related characteristics that influence prognosis and must be considered when choosing the most appropriate treatment. Therapeutic decisions must be based, not only on the tumor’s characteristics, but also on the patient’s physical, mental, and social ability to tolerate treatment [5], underlining the importance of multidimensional, multidisciplinary assessment.

The European Society for Medical Oncology’s consensus document for the management of older patients with lymphoma focuses on several key aspects [6], including identification of frail patients and individualized treatment. While robust older patients may be eligible for curative treatment at full doses, frail patients with comorbidities may require treatment modifications. Developing robust strategies to detect vulnerable patients at risk of complications is the key to personalizing treatment for older HM patients. Comprehensive geriatric assessment (CGA) has proven its efficacy in aiding treatment decision-making and guiding outcomes in different studies [7–10], and uses specific instruments to identify patient frailty, which confers increased vulnerability to adverse events (disability, hospitalization, and death). The International Society of Geriatric Oncology (SIOG) and National Comprehensive Cancer Network (NCCN) consensus guidelines recommend the use of CGA in all older cancer patients [11, 12].

Despite the relevance of CGA for older patients diagnosed with cancer, to the best of our knowledge, there are no studies reporting large series of older patients with lymphoma who have undergone standardized CGA for personalized oncospecific treatment. Our study, carried out in a tertiary care hospital (Madrid, Spain), presents the application of a systematic protocol for specialized CGA in patients with lymphoma over 70 years of age, allowing frailty-based patient classification, individualized care recommendations, and treatment personalization. We also report patient outcomes after the start of oncospecific treatment.

## Methods

Lymphoma patients over 70 years of age referred to the Geriatric Hematology clinic at the Fundación Jiménez Díaz University Hospital (Madrid, Spain) for specialized CGA between May 1st, 2016, and March 31st, 2021, were included in the study. All patients presented a recent diagnosis of lymphoma and had been approved for oncospecific treatment by the hospital’s tumor committee. Patients undergoing second or subsequent lines of treatment were excluded. No other exclusion criteria were defined.

Patients approved for oncospecific therapy were referred to the geriatric hematology clinic as a part of our clinical pathway for assessment using a systematic CGA protocol. Evaluation was carried out by a qualified geriatrician with broad experience in geriatric oncology and hematology. This assessment was usually performed 1–2 weeks before starting oncospecific treatment. Neither patient classification nor subsequent interventions delayed the start of treatment.

The CGA protocol included the domains recommended by Mohile et al. [13]: assessment of comorbidity; presence of polypharmacy; nutritional, functional, and mental status; physical performance tests; life expectancy; and the presence of geriatric syndromes such as urinary or fecal incontinence, falls, or history of depression or dementia. Information was stored and retrieved for analysis from the hospital’s electronic health record, Casiopea® (Inetum).

Socio-demographic variables (age, sex, and living arrangements (alone or accompanied)) and Eastern Cooperative Oncology Group (ECOG) [14] performance status were collected. The Barthel Index [15] (Basic activities of daily living), the Lawton and Brody Index [16] (Instrumental activities of daily living) and the FAC [17] (Functional Ambulation Categories) scale for mobility were used to assess functional status. Mental status was assessed using the Pfeiffer Short Portable Mental Status Questionnaire (SPMSQ) [18]. The Global Deterioration Scale [19] (GDS) was used to describe patients’ cognitive status, and the Yesavage scale [20] to assess the presence of depression. Previous diagnoses of depression were also recorded. Nutritional screening was carried out using the Mini Nutritional Assessment Short Form [21] (MNA-SF) and body mass index (BMI). Comorbidity was assessed using the Cumulative Illness Rating Scale-Geriatric [22] (CIRS-G). Polypharmacy was defined as the simultaneous prescription of 5 or more drugs. Life expectancy was calculated based on the patient’s medical history and baseline situation, using the ePrognosis application (www.eprognosis.com). A score equal to or greater than 12 predicted 5-year mortality. Analytical parameters such as albumin, lactate dehydrogenase (LDH) and hemoglobin values were also recorded. The Short Physical Performance Battery [23] (SPPB) and the FRAIL [24] questionnaire were used to assess frailty. Assessment tools were selected based on usual geriatric practice and on past research on geriatric oncology carried out by our team.

Data were manually extracted from the electronic health record, including: type and characteristics of the different lymphomas, including cell of origin, grade, lymphoma subtype, revised international prognostic index (R-IPI) [25], presence or absence of B symptoms, and extranodal involvement; oncospecific treatment data, including start and end date, and treatment modifications; and treatment results, including tolerance, need for treatment modifications, reason for modification, toxicity, and severity (grade > 2)). Severity of adverse events was evaluated using the Common Terminology Criteria for Adverse Events (CTCAE) [26]. Response to treatment, disease progression, relapse, and the date of relapse when applicable were also recorded.

Interventions implemented by the geriatrician after CGA, including nutritional interventions, physical activity recommendations, antidepressant prescriptions, polypharmacy reduction strategies, and social interventions were recorded. Information on patient mortality and use of health resources (number of emergency room visits and number of hospital admissions) during the year after starting treatment was also collected.

### Intervention model: comprehensive assessment, patient classification, and multidisciplinary approach

Comprehensive geriatric assessment enabled patient classification using a modification of the criteria proposed by Balducci and Extermann [27, 28] based on frailty assessment instrument scoring and clinical, functional, and mental status. Four groups were identified: Type I (“fit” patients with no detected deficits); Type II (“pre-frail” patients with potentially correctable deficits); Type III (“frail” patients, with irreversible deficits); and Type IV (disabled patients and those with a poor overall prognosis). A similar classification has been used in a previous study [29].

With the aim of optimizing patients’ health status before, during, and after treatment, and providing personalized care throughout the therapeutic process [30–32], the geriatrician conducting CGA made specific recommendations for each of the deficits or problems detected during assessment. Patient-tailored recommendations included nutritional advice, prescription of specific physical exercise using the VIVIFRAIL program (web.vivifrail.com) [33], adjustments in polypharmacy (according to the STOPP/START criteria [34]), and control of cardiovascular risk factors. Patients were scheduled for follow-up appointments every 3 months during the first year of treatment, or more frequently if needed. We collected data from the follow-up appointments to detect increased frailty (measured using functional scales or the appearance of new geriatric syndromes). At follow-up appointments, only functional tests (the Barthel Index and Lawton Index) were performed.

After CGA and patient classification had been carried out, the hospital’s multidisciplinary lymphoma committee, which includes hematologists, pathologists, nuclear medicine specialists, radiologists, hospital pharmacists, and geriatricians, selected oncospecific treatment for each patient. Type I (“fit”) patients were prescribed standard oncospecific treatment; patients classified as types II, III and IV were prescribed adapted regimens featuring lower doses, longer intervals between cycles, and drugs with lower risk of cardiotoxicity.

### Statistical analysis

Qualitative variables were presented as frequencies and percentages, and quantitative variables as mean and standard deviation or median and quartiles, depending on distribution. Comparisons of qualitative variables were performed using Pearson’s chi-squared test or Fisher’s exact test. Comparisons of quantitative variables were performed using one-way ANOVA or Student’s *t* test for those variables summarized as mean and standard deviation, and Kruskal–Wallis or Wilcoxon’s rank test for those variables summarized as median and quartiles. Overall survival (OS) time was computed from the date of diagnosis to either the date of the last visit that the patient was known to be alive or the date of death from any cause. Survival curves for each group of patients were estimated using the Kaplan–Meier method and compared using a log-rank test. Multivariate analysis of survival was performed using the Cox proportional hazard ratio (HR) with a 95% confidence interval (CI), considering all variables that had been shown to be significantly associated with survival in the univariate analysis. Statistical analyses were performed using R version 4.1.2 (R: A language and environment for statistical computing. R Foundation for Statistical Computing, Vienna, Austria).

The study was approved by the Hospital Clinical Research Ethics Committee of the Fundación Jiménez Díaz University Hospital (EO121-21_FJD).

## Results

Between May 1st, 2016, and March 31st, 2021, 93 patients aged 70 years and above with a recent diagnosis of lymphoma underwent CGA at our hospital. Median age at assessment was 81.1 years (± 5.7 years), and 55.9% of patients were women. The majority of lymphomas diagnosed were NHL (94.5%), high-grade (66.7%), and B-cell (91.4%). The most frequent lymphoma subtype was diffuse large B-cell lymphoma (48.4%), followed by marginal (10.8%) and follicular (9.7%) lymphoma. Most patients presented a high R-IPI (51.6%) and extranodal involvement (75.3%) and did not present B symptoms (55.9%). Most patients were assessed before starting oncospecific treatment. Only 20% (mainly patients with diffuse large B-cell lymphoma) were assessed after having received prephase treatment or the first cycle of chemotherapy.

Regarding classification, 23 patients (24.7%) were classified as robust (type I), 30 patients (32.3%) as pre-frail (type II) with potentially reversable deficits, 38 patients (40.9%) as frail (type III), and only 2 patients (2.2%) as presenting with a poor overall prognosis or requiring palliative care (type IV). Type III and IV patients were analyzed as a group. This decision was made after data collection, because only two type IV patients were identified, which made comparison with other groups impossible.

Patients’ clinical characteristics, and the differences between groups, are presented in Table 1. We observed significant differences regarding comorbidity across groups, with a higher CIRS-G score in type III–IV patients (*p* 0.006). Likewise, functional status was worst in the type III–IV group, with lower overall Barthel Index (*p* 0.005), FAC (*p* < 0.001) and Lawton Index (*p* < 0.001) scores. Higher GDS (*p* 0.008) and lower Pfeiffer questionnaire scores (*p* 0.034) in the type III–IV group indicated significantly worse cognitive status compared to other groups. Significant differences in frailty scores (SPPB (*p* < 0.001) and FRAIL questionnaire (*p* 0.002)) and analytical parameters (hemoglobin (*p* 0.021), albumin (0.006)) were also observed, with patients in the type III–IV groups showing increased frailty and lower hemoglobin and serum albumin levels.

**Table 1:** Baseline characteristics of lymphoma patients included in a geriatric oncohematologic program and their classification (type I, type II and type III–IV) according to Comprehensive Geriatric Assessment (CGA)

Variable	Type I (*n* 23)	Type II (*n* 30)	Type III–IV (*n* 40)	*P*
Age	78.3 ± 4.6	79.3 ± 4.4	84.1 ± 5.8	< 0.001
Sex				0.988
Woman	13 (56.5%)	17 (56.7%)	22 (55%)	
ECOG				0.004
Fully active	13 (59.1%)	11 (36.7%)	9 (22.5%)	
Restricted in physically strenuous activity	8 (36.4%)	15 (50.0%)	14 (35.0%)	
Ambulatory and capable of all self-care, up more than > 50%	1 (4.5%)	4 (13.3%)	10 (25.0%)	
Capable of only limited self-care, bed or chair > 50%	0 (0.0%)	0 (0.0%)	7 (17.5%)	
CIRS-G total	5.6 ± 3.7	6.6 ± 3.3	9 ± 4.4	0.006
Polypharmacy (≥ 5 drugs)	10 (43.5%)	19 (65.5%)	28 (71.8%)	0.078
Barthel index				0.005
Independent	17 (73.9%)	19 (63.3%)	13 (32.5%)	
Lawton index	6.9 ± 1.6	5 ± 2.3	2.9 ± 2.1	< 0.001
Independent walking (FAC: 5)	22 (95.7%)	21 (72.4%)	15 (37.5%)	< 0.001
FRAIL questionnaire (≥ 3)	3 (13.0%)	16 (55.2%)	28 (71.8%)	< 0.001
SPPB < 10	9 (40.9%)	14 (60.9%)	27 (87.1%)	0.002
Mini nutritional assessment ≤ 11	3 (13.6%)	8 (32.0%)	17 (50.0%)	0.019
Body mass index	26.4 ± 4	25.6 ± 4.4	26.6 ± 4.7	0.669
Pfeiffer questionnaire > 2	0 (0.0%)	0 (0.0%)	6 (17.1%)	0.008
Global deterioration scale > 2	1 (4.3%)	1 (3.3%)	8 (21.1%)	0.034
Living alone	6 (26.1%)	6 (20.0%)	3 (7.5%)	0.371
Presence of geriatric syndrome	11 (47.8%)	10 (33.3%)	26 (65.0%)	0.031
Previous falls	0 (0.0%)	0 (0.0%)	4 (10.0%)	0.086
Urinary or fecal incontinence	4 (17.4%)	3 (10.0%)	21 (52.5%)	< 0.001
Previous depression	3 (13.0%)	2 (6.7%)	6 (15.0%)	0.553
ePrognosis ≥ 12	1 (10.0%)	4 (22.2%)	14 (60.9%)	0.006
Subtype lymphoma				0.340
Follicular	5 (21.7%)	1 (3.3%)	3 (7.5%)	
DLBCL	9 (39.1%)	17 (56.7%)	19 (47.5%)	
Mantle cell lymphoma	2 (8.7%)	0 (0.0%)	1 (2.5%)	
Hodgkin’s lymphoma	1 (4.3%)	1 (3.3%)	3 (7.5%)	
Lymphocytic lymphoma	2 (8.7%)	1 (3.3%)	4 (10.0%)	
Lymphoma T	1 (4.3%)	4 (13.3%)	3 (7.5%)	
Marginal	2 (8.7%)	5 (16.7%)	3 (7.5%)	
R-IPI				0.591
Low	4 (17.4%)	3 (10%)	3 (7.5%)	
Intermediate	9 (39.1%)	9 (30%)	17 (42.5%)	
High	10 (43.5%)	18 (60%)	20 (50%)	
LDH > 250 UI/L	20 (87.0%)	20 (66.7%)	24 (61.5%)	0.101
Albumin ≤ 3.5 g/dl	1 (4.3%)	8 (26.7%)	14 (35.9%)	0.021
Hemoglobin < 12 g/dl	5 (21.7%)	17 (56.7%)	25 (62.5%)	0.006

Data are presented as *n* (%), except age, CIRS-G, Lawton Index, MNA and IMC, which are expressed as mean (SD)

*ECOG* indicates Eastern Cooperative Oncology Group, *CIRS-G* Cumulative Illness Rating Scale-Geriatric, *FAC* Functional Assessment Categories, *SPPB* Short Physical Performance Battery, *DLBCL* indicates diffuse large B-cell lymphoma B, *R-IPI* International prognostic index review

No significant differences regarding lymphoma subtypes and clinical characteristics were found between groups. Treatment was chosen on a tailored basis, considering patients’ values and preferences, by the hospital’s multidisciplinary lymphoma tumor board. Oncospecific treatment differed significantly across groups (*p* < 0.001), with adapted regimens (64.5%) being more frequent in type II and III–IV patients (*p* 0.001) (Table 2). The rate of subsequent treatment modifications did not vary between groups. 14% of patients discontinued treatment due to side effects, with similar rates of severe (> grade 2) toxicity and discontinuation due to toxicity observed across groups. There were no differences regarding the need for subsequent treatment adjustments (modification or discontinuation of the initial treatment). With regards to geriatric intervention (Table 3), nutritional recommendations were given to 82.2%, oral nutritional supplements were prescribed to 39.8%, and an individualized physical exercise program was prescribed to 59.1% of patients. General exercises were recommended to all, but 60% of patients were prescribed specific, individualized exercise using the VIVIFRAIL program.

**Table 2:** Differences in oncospecific treatment according to patient classification as type I, type II, and types III–IV according to results of comprehensive geriatric assessment

Variable	Type I (*n* 23)	Type II (*n* 30)	Type III–IV (*n* 40)	*p*
*Treatment*				< 0.001
Chop like	12 (52.2%)	11 (36.7%)	2 (5%)	
Mini-chop	1 (4.3%)	12 (40%)	18 (45%)	
Bendamustine-Rituximab	3 (13%)	2 (6.7%)	3 (7.5%)	
Palliative	0 (0%)	1 (3.3%)	8 (20%)	
Others	5 (21.7%)	3 (10%)	7 (17.5%)	
Rituximab	2 (8.7%)	1 (3.3%)	2 (5%)	
*Treatment*				0.001
Standard	13 (56.5%)	14 (46.7%)	6 (15%)	
Adapted	10 (43.5%)	16 (53.3%)	34 (85%)	
*Change of treatment after initial decision*				0.934
No	16 (69.6%)	20 (66.7%)	26 (65%)	
Yes	7 (30.4%)	10 (33.3%)	14 (35%)

**Table 3:** Specific geriatric interventions prescribed

Variable	*n* (%)
Nutritional recommendations	77 (82.7%)
Specific exercise recommendations	55 (59.2%)
CVRF control	44 (47.3%)
Prescription of oral nutritional supplements	37 (39.8%)
Social intervention	17 (28.3%)
Prescription of antidepressant treatment	8 (8.6%)

Data presented in *n* (%)

*CVRF* indicates cardiovascular risk factors

**Fig. 1:**
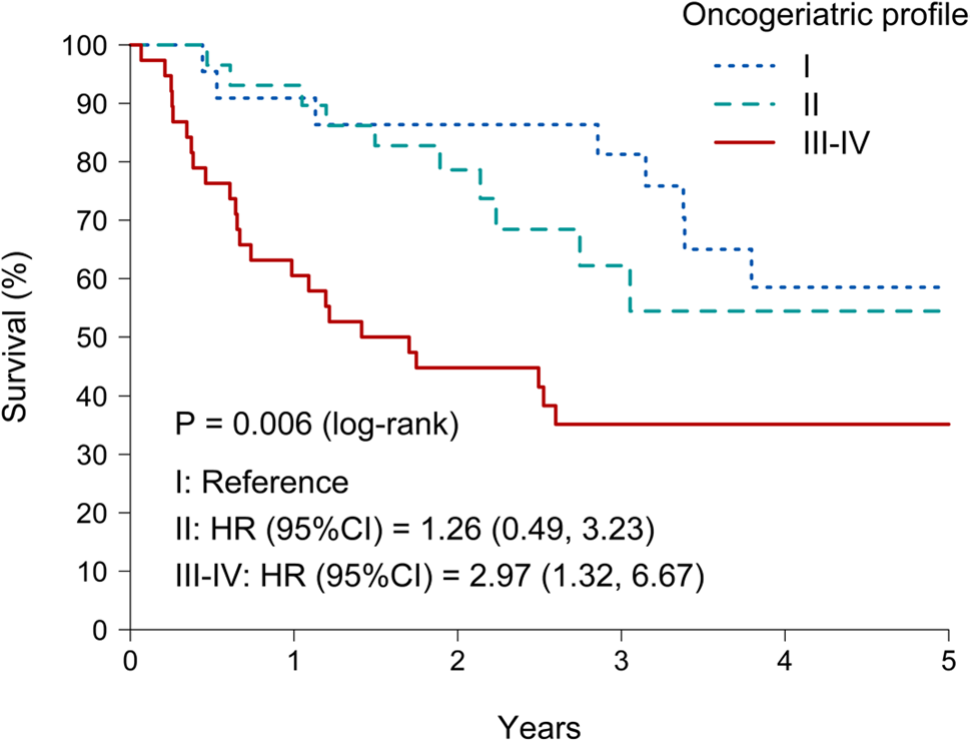
Kaplan–Meier-estimated overall survival curves for patients type I, II and III–IV according to comprehensive geriatric assessment

Regarding response to treatment, overall survival, and frailty at the end of treatment (Table 4), significant differences were observed. 53.8% of patients achieved complete response at the end of treatment, 34.4% achieved partial response, 9.7% showed no response to treatment, and 2.2% died during treatment (2 patients, one from the type I group and another from the type III–IV group). At follow-up (median follow-up 27.3 months, range 18–74 months), 25.8% patients presented a relapse, with no differences between groups. Survival analysis was performed using Kaplan–Meier curves (Fig. 1); we found that fit (type I) patients presented higher survival rates compared to those in the type III–IV group (42.5 ± 19.6 months versus 23.7 ± 20.5 months, *p* 0.002). Increased frailty at the end of treatment during the follow-up period was much less frequent in type I patients compared with type III–IV patients (9.1% vs 39.5%, *p* 0.024). No differences were observed regarding the use of hospital resources, including hospital admissions and emergency department visits, during the year after starting treatment.

**Table 4:** Clinical outcomes of patients classified as type I, type II, and types III–IV according to results of comprehensive geriatric assessment

Variable	Type I (*n* 23)	Type II (*n* 30)	Type III–IV (*n* 40)	*P*
*Treatment response*				0.001
Extended disease without response	1 (4.3%)	1 (3.3%)	7 (17.5%)	
Mortality during treatment	1 (4.3%)	0 (0.0%)	1 (2.5%)	
Complete response	14 (60.9%)	24 (80%)	12 (30%)	
Partial response	7 (30.4%)	5 (16.7%)	20 (50%)	
Severe toxicity	7 (31.8%)	6 (20%)	10 (25.6%)	0.624
Mortality during follow-up	9 (40.9%)	11 (37.9%)	24 (64.9%)	0.058
Relapse	7 (31.8%)	9 (30%)	8 (20.5%)	0.541
Frailer at the end of the treatment	2 (9.1%)	6 (20%)	15 (39.5%)	0.024
Overall survival (months)	42.5 ± 19.6	29.7 ± 17.2	23.7 ± 20.5	0.002
Disease-free survival (months)	20.6 ± 19.8	14.2 ± 8.1	10.4 ± 7.9	0.403
Treatment duration (months)	8.40 ± 12.9	5.7 ± 6.3	4.5 ± 4.1	0.310

We performed univariate and multivariate analyses using a COX regression model to identify predictors of mortality (Table 5). As expected, age and moderate-to-severe dependence measured using the Barthel Index were found to correlate to mortality, while higher LDH levels (< 250 mg/dL) and the presence of geriatric syndromes almost reached statistical significance (*p* = 0.076 and *p* = 0.062, respectively). The regression model showed acceptable goodness of fit, with a C-statistic of 0.70.

**Table 5:** Uni- and multivariate analyses of comprehensive geriatric assessment, treatment, and interventions on overall survival

Variables	Univariate HR (CI 95%)	*P*	Multivariate HR (CI 95%)	*P*
Age	1.08 (1.02,1.14)	0.005	1.07 (1.01–1.14)	0.020
Age > 80 years	2.03 (1.05, 3.92)	0.034		
LDH	1.03 (1.00, 1.06)	0.050	1.03 (1.00–1.06)	0.076
Presence urinary/fecal incontinence	1.91 (1.03, 3.54)	0.040		
Treatment (adapted)	2.85 (1.32, 6.17)	0.008		
Taking oral nutritional supplements	1.88 (1.02, 3.45)	0.043		
Barthel index (moderate-severe dependence)	4.45 (2.12,9.34)	< 0.001	4.16 (1.94–8.93)	0.001
Patient group (type III–IV)	2.62 (1.42, 4.85)	0.002		
Presence of geriatric syndromes	1.77 (0.94, 3.34)	0.077	1.87 (0.97–3.63)	0.062

*HR* indicates hazard ratio, *CI* confidence interval

## Discussion

This study presents a cohort of older patients with NHL assessed with a systemic CGA protocol to enable individualized oncospecific treatment. The goal of our research was to determine if the application of a systemic CGA protocol in older patients with a recent diagnosis of lymphoma could enable patient classification according to frailty profiles, prescription of geriatric care recommendations, and tailored oncospecific treatment. We also aimed to describe the impact of CGA on clinical outcomes.

CGA yielded exhaustive information on patients’ functional capacity, comorbidity, level of frailty, nutritional status, cognitive status, geriatric syndromes, and estimated survival. These data allowed us to classify patients into three groups (type I, II, and III–IV). Although no differences were observed regarding the types of lymphoma diagnosed across groups, patient classification permitted individualized care, including personalized geriatric recommendations to improve nutritional status, physical condition, and cardiovascular risk factors, as well as frailty-based adaptation of oncospecific treatments. During follow-up, toxicity rates were similar for the different groups, and no differences in the use of hospital resources were observed, leading us to consider that initial treatment had been chosen appropriately. Despite the use of more intensive regimens in groups I–II, no increase in hospitalization rates and emergency room visits was observed. However, mortality rates and frailty among surviving patients were significantly higher in the type III–IV group.

Previous attempts have been made to classify older patients with lymphoma according to non-hematologic characteristics. However, there is no definitive consensus on the most appropriate instruments, scores, or scales for classification. Some studies (most of which have been performed in an older population with diffuse large B-cell lymphoma) have identified a series of prognostic factors associated with worse clinical outcomes and lower survival, using domains such as functional impairment, dependence for basic or instrumental activities, presence of malnutrition and comorbidity [36, 37]. Tools focusing on these domains are capable of identifying frailty more accurately than clinician judgment or performance status (PS) alone [38].

Existing studies seeking to predict unfavorable outcomes in older patients with lymphoma include research published by the Italian Lymphoma Foundation [39] (FIL), featuring a simplified geriatric assessment including basic and instrumental activities of daily living, comorbidity, and age, and Miura et al.’s study [40] describing the development of the ACA Index to predict outcomes using age, comorbidity, and albumin blood levels. Liu et al. [41] combined the ACA index with an assessment of functional status (IADL) to create the IADL-ACA (IACA) index for patients ≥ 65 years of age with diffuse large B-cell lymphoma, allowing patient classification into three risk groups—low, intermediate, and poor—observing significant differences in overall response rate, cumulative incidence of treatment-related mortality, relapse rate, and 2-year overall survival. Although our study did not aim to create a predictive score, we were able to detect different areas that can help to classify older patients with lymphoma and that could potentially serve as a basis for the creation of predictive models using a larger series.

Two aspects of our study should be highlighted. On one hand, the detection of frailty through CGA revealed unmet needs for geriatric intervention to improve patients’ overall health status. These interventions (physical exercise programs, nutritional support, and psychological interventions) have also been carried out in other cancer settings and have been described as positively influencing patient outcomes [42, 43]. On the other hand, the information gathered through CGA and patient classification allowed the lymphoma committee to tailor treatment for each patient. To the best of our knowledge, this is the first study reporting systematic individualization of oncospecific treatment according to frailty status. For example, in Tucci’s study [39], patients were treated according to the attending physician’s clinical judgement regardless of category, while Garrick et al. [44] report that frailty had a slight influence on the choice of treatment, leading to a change of treatment in only 21.7% of cases.

Adjusting treatment according to patients’ characteristics allowed us to achieve a higher percentage of complete responses in type I and II patients, similar to that observed in younger populations (60–80% 5-year complete response rates, depending on the subtype of lymphoma), without increasing toxicity, use of health resources, or need for treatment. These results indicate appropriate choice of initial treatment by the lymphoma committee. Other studies, such as Corre et al. [45], have demonstrated similar results in advanced non-small-cell lung cancer, with CGA-based individualized treatment failing to improve treatment outcomes but slightly reducing treatment toxicity. Mohile et al. [46] report that older patients with advanced cancer undergoing CGA (incurable solid tumors or lymphoma) experience less grade 3–5 toxicity than their non-CGA counterparts. These studies highlight the importance of CGA-guided interventions to improve outcomes, although more specific studies are needed to determine how CGA-tailored treatment can reduce toxicity for older individuals with lymphoma.

Strengths of this study include the thoroughness with which geriatric assessment was performed, and the close clinical follow-up patients received during treatment. We believe that CGA carried out by an expert physician, instead of using standalone frailty scales, is one of the greatest strengths of the study. In our opinion, CGA-mediated patient selection enabled the lymphoma committee to carry out comprehensive evaluation and therapeutic decision-making, while geriatric intervention during oncospecific treatment played an important role in the study’s results. In routine care, re-performing frailty assessments after the start of an oncological treatment together with non-oncological frailty interventions would allow us to assess whether and to what degree a patient is responsive to such management. This would enable a multidisciplinary team to decide whether frailty interventions should be continued, escalated, de-escalated, or stopped. Furthermore, frailty monitoring over time may guide hematologists to increase or decrease the intensity of oncospecific treatment. To date, however, no studies have explored the utility of repeated frailty assessments to guide continuous adaptation of ongoing cancer treatment, and how to perform re-assessment [35].

One of our study’s limitations is the lack of a control group, which could have helped us to understand the implications of this care strategy better. Moreover, we cannot draw robust conclusions regarding different lymphoma subtypes due to the small sample size. To overcome these limitations, prospective randomized trials using CGA as a stratum criterion should be planned. We expect future studies to validate the efficacy of CGA-based therapy across different lymphoma subgroups.

In conclusion, the oncohematogeriatric approach to care using CGA enables geriatric intervention in older patients with lymphoma, classifies patients according to their frailty status, and aids the decision-making process by allowing individualized treatment tailored to patients’ overall condition and personal preferences. Our results reinforce the value of multidisciplinary teams that include geriatricians to personalize oncospecific therapy according to the clinical, functional and frailty status of each patient. This study is one of the first to demonstrate oncohematogeriatric assessment and intervention and its influence on treatment outcomes.

We propose incorporating a CGA protocol and ensuring the presence of geriatricians as part of a multidisciplinary care team as part of the optimal therapeutic strategy for older patients with lymphoma. If multidisciplinary or geriatric inputs are not available, it is important to design a predefined intervention plan [46] for these patients. Moving forward, there is a need for further studies on the role of CGA regarding prognosis and management of older adults with lymphoma. Future randomized studies should focus on providing evidence for optimal therapeutic options guided by geriatric assessment [47].

## Data Availability

The datasets generated and/or analyzed in the current study are not publicly available, but available from the corresponding author upon reasonable request.
